# Supplementary material for: Computed Tomography Angiography for Detection of Pulmonary Embolism in Western Australia Shows Increasing Use with Decreasing Diagnostic Yield
Source: J Clin Med. 2023 Jan 27;12(3):980. doi: 10.3390/jcm12030980 (PMC9917579; doi:10.3390/jcm12030980)

Supplementary file

Table S1: Full output of logistic regression assessing diagnostic yield. The cohort is made up of people with an unplanned emergency department (ED) presentation at a tertiary hospital with associated computed tomography pulmonary angiography (CTPA) procedure. The dependent variable is pulmonary embolism (PE) diagnosis, indicated by hospital admission with PE as principal diagnosis the day of / after the CTPA.

**Independent var**		**OR**	**SE**	**Z**	**P>|z|**	**Lower CI**	**Upper CI**
Year	2003	1.05	0.27	0.19	0.849	0.63	1.74
	2004	1.12	0.30	0.44	0.663	0.67	1.89
	2005	1.53	0.26	2.51	0.012	1.10	2.12
	2006	1.49	0.23	2.56	0.010	1.10	2.01
	2007	1.15	0.17	0.95	0.340	0.86	1.52
	2008	1.28	0.17	1.84	0.065	0.98	1.66
	2009	1.46	0.17	3.16	0.002	1.15	1.85
	2010	1.06	0.13	0.46	0.648	0.84	1.33
	2011	1.00	0.12	0.01	0.992	0.80	1.25
	2012	0.98	0.11	-0.22	0.827	0.78	1.21
	2013	0.89	0.10	-1.05	0.296	0.71	1.11
	2014	1.07	0.11	0.62	0.535	0.87	1.31
	2015	Reference					
Sex	Female	0.74	0.04	-5.39	0.000	0.66	0.82
Socioeconomic status	Highest disadv.	Reference					
	High disadv.	1.01	0.08	0.07	0.946	0.86	1.18
	Moderate disadv.	1.06	0.09	0.63	0.528	0.89	1.25
	Less disadv.	1.12	0.10	1.24	0.215	0.94	1.33
	Least disadv.	1.24	0.11	2.33	0.020	1.03	1.48
	Unknown	0.56	0.28	-1.18	0.238	0.21	1.47
Remoteness	Major cities	Reference					
	Inner regional	1.15	0.13	1.16	0.248	0.91	1.44
	Outer regional	0.85	0.14	-1.01	0.311	0.61	1.17
	Remote	2.09	0.52	2.97	0.003	1.29	3.39
	Very remote	0.72	0.17	-1.39	0.163	0.46	1.14
	Unknown	0.95	0.51	-0.10	0.919	0.33	2.74
Triage code	Resuscitation	1.47	0.23	2.40	0.016	1.07	2.01
	Emergency	Reference					
	Urgent	1.22	0.08	3.13	0.002	1.08	1.38
	Semi urgent	1.18	0.13	1.58	0.114	0.96	1.46
	Non urgent	0.54	0.29	-1.16	0.247	0.19	1.52
Arrival type	Private vehicle	Reference					
	Ambulance	1.10	0.06	1.70	0.090	0.98	1.24
	Other	1.08	0.30	0.26	0.796	0.62	1.87
	Self	Reference					

**Independent var**		**OR**	**SE**	**Z**	**P>lzl**	**Lower CI**	**Upper CI**
Referral source	GP - Letter	1.29	0.20	1.67	0.096	0.96	1.75
	GP - no letter	1.68	0.48	1.83	0.067	0.96	2.94
	Clinic	1.82	0.74	1.47	0.140	0.82	4.03
	Other hospital	0.46	0.13	-2.74	0.006	0.27	0.80
	Other	1.18	0.13	1.53	0.126	0.95	1.46
	Unknown	1.10	0.15	0.73	0.466	0.85	1.43
Constant		0.18	0.19	0.02	-13.21	0.000	0.15

age omitted due to non-significance

Table S2: Diagnostic yield of CTPA performed in the ED by year. Margins represent adjusted probabilities of PE hospitalisation following CTPA, generated following the regression detailed in Table S1.

	**Year**	**Margin**	**SE**	**z**	**P>z**	**Lower CI**	**Upper CI**
	2003	0.13	0.03	4.68	<0.001	0.07	0.18
	2004	0.13	0.03	4.57	<0.001	0.08	0.19
	2005	0.17	0.02	8.06	<0.001	0.13	0.22
	2006	0.17	0.02	8.98	<0.001	0.13	0.21
	2007	0.14	0.01	9.46	<0.001	0.11	0.17
	2008	0.15	0.01	10.82	<0.001	0.12	0.18
Year	2009	0.17	0.01	13.02	<0.001	0.14	0.19
	2010	0.13	0.01	12.37	<0.001	0.11	0.15
	2011	0.12	0.01	13.11	<0.001	0.10	0.14
	2012	0.12	0.01	13.48	<0.001	0.10	0.14
	2013	0.11	0.01	13.45	<0.001	0.09	0.13
	2014	0.13	0.01	15.46	<0.001	0.11	0.15
	2015	0.12	0.01	15.45	<0.001	0.11	0.14

Figure S1: Comparison of the age-sex standardised rate of hospitalisation for cerebrovascular disease in WA and rates of death during cerebrovascular hospitalisation

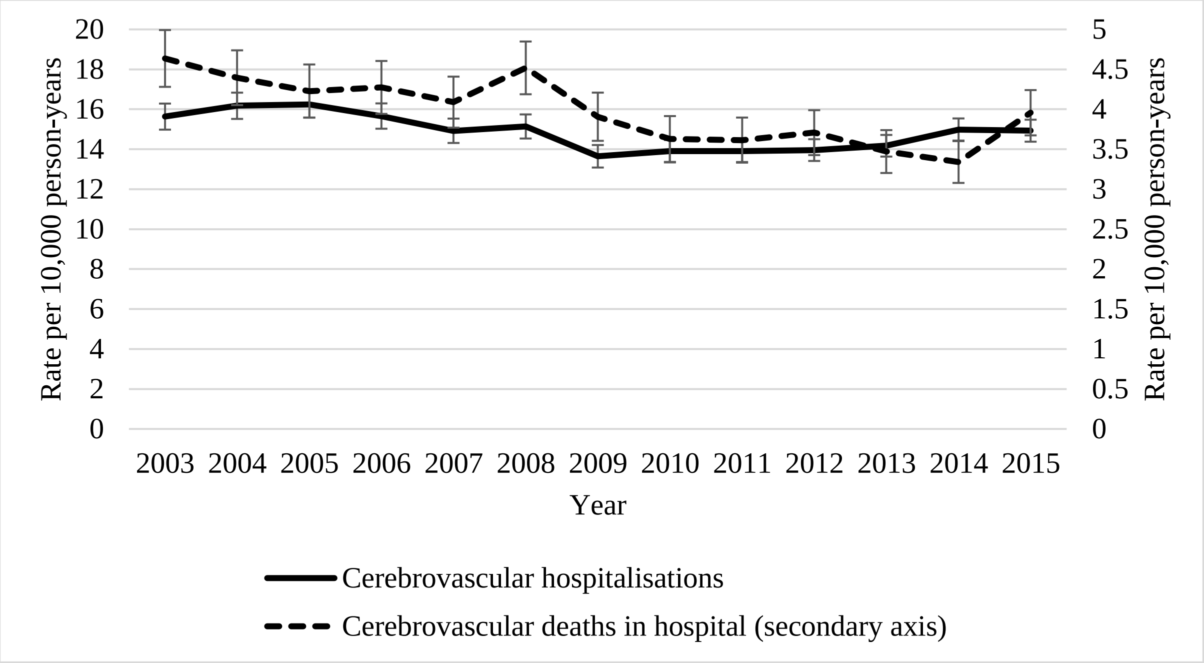

Supplement: Supplementary file 1 [file jcm-12-00980-s001.zip › jcm-2129001-supplementary.pdf]
